# Inferring the energy sensitivity and band gap of electronic transport in a network of carbon nanotubes

**DOI:** 10.1038/s41598-022-06078-x

**Published:** 2022-02-08

**Authors:** Shuang Tang

**Affiliations:** College of Engineering, State University of New York Polytechnic Institute, Albany, NY 12203 USA

**Keywords:** Chemistry, Energy science and technology, Engineering, Nanoscience and technology

## Abstract

Since the industrialization of single-phase nanomaterial-based devices is still challenging, intensive research focus has been given to complex materials consisting of multiple nanoscale entities, including networks and matrices of nanowires, nanotubes, nanoribbons, or other large molecules; among these complex materials, networks of carbon nanotubes are a typical example. Detailed knowledge of the energy sensitivity and band gap of electronic transport in such a material system is difficult to detect, despite its importance in electronic, energetic and sensing applications. Here, we propose a new methodology to obtain these quantities using the measured Seebeck coefficient at a certain temperature but different Fermi levels. We discover that the network consisting of semiconducting *(11,10)*-carbon nanotubes actually exhibits metallic transport at room temperature. It is also interesting to verify that intrananotube ballistic transport is dominant over diffusive scattering by long-range disorder, as well as the quantum hopping resistance at the contact points. The transport asymmetry ratio between the holes and electrons (1.75) is similar to the value observed in pristine graphene samples (1.50).

## Introduction

Modern nanotechnology has enabled the laboratory study of single-phase nanoscale and/or molecular materials, including quantum dots^1^, nanowires^2^, various organic and biological molecules^3,4^, and others^5,6^. However, manufacturing devices utilizing such single-phase entities on the industrial scale remains a challenge. Multiphase nanostructured materials assembled of multiple individual nanoscale entities, including networks^7,8^, matrices^9^, and mosaic thin films^10^ of nanowires^11^, nanotubes^12^, nanoribbons^13^, and protein or DNA molecules^14,15^, have attracted intensive research interest, and a network of carbon nanotubes is a typical example^16–19^.

For realization and improvement of electronic and energetic applications, it is important to understand and harness the transport behaviors of charge carriers in carbon nanotube networks and other nanostructured materials. Under external force fields, the charge carriers will collectively move and transfer energy, entropy and information through diffusive^20^, ballistic^21–23^, and quantum hopping transport^24,25^. Scatterings to the carriers will result from disorders induced by phonons^26^, ionized impurities^27^, point contacts^28^, and many other imperfections^26,29^. Under stable conditions, the external force field will stay in equilibrium with the scattering centers, and the carriers will be transported at a certain rate (*θ*)^30^, which describes the average net concentration of carriers passing through a unit cross-sectional area per second. This transport rate generally changes with the carrier energy. However, the energy sensitivities of carrier transport in carbon nanotube networks have not yet been measured by experiments. The lack of such energy sensitivity information hinders the development of related applications such as electronic filters and Bloch wave prisms.

The energy dispersion relation for carriers in carbon nanotube networks also has larger uncertainty than single carbon nanotubes. In a single carbon nanotube, the energy band gap and energy dispersion of carriers are well determined by the chirality^31^. However, in a carbon nanotube network, the energy dispersion, as well as the band gap, of carriers will be influenced by the internanotube contact modes, the nanotube diameter distribution, impurities and others^32,33^. It is also found that the energy band gap of a carbon nanotube network can be significantly smaller than that of a single carbon nanotube^34,35^. The electrical conductivity and capacitance of carbon nanotube networks as a function of gate voltage are measured^36–40^, which can be used to obtain an intuitive understanding of the transport band gap. However, it is still a challenge to quantitatively infer the transport band gap only from the measured electrical conductivity, e.g., the permeability of the dielectric layer might be inaccurately estimated, and the thermal energy can cause the band edges to be smeared at the extent of several *k*_*B*_*T*s.

We have discovered that the Seebeck coefficient, a less frequently used transport quantity, can be used to extract a set of electronic features depicting the statistical details of electron and hole transport in a material system, with diffusive, ballistic and/or quantum tunneling transport mechanism(s)^41^. The effective band gap, electron–hole asymmetry, and energy sensitivity to carrier scattering/transport can all be inferred^42^.

This paper will focus on exploring the inference of energy sensitivity for carrier transport in a complex network thin film consisting of individual carbon nanotubes. We will first describe our new methodology of inferring such energy sensitivities using the highest achievable Seebeck coefficient, then apply it to ideal carbon nanotube networks with a high purity of chirality and examine how the energy sensitivity determines the Seebeck coefficient. Furthermore, we will apply the methodology to the experimental data measured for a thin film sample of a *(11,10)*-carbon nanotube network. The metallicity, scattering mechanisms, and transport regimes are discussed. Finally, we suggest that this new method be used in other networks or matrix systems of nanotubes, nanowires, and large molecules.

## Methodology

Intuitively, the scattering rate (*θ*) describes the concentration of carriers that have passed through a cross-sectional area within one unit time interval^30^. The transport rate will generally change corresponding to the change in carrier energy. The energy sensitivity of transport can then be defined as the relative transport rate change per relative carrier energy change^42^, i.e., *s* = (*dθ/θ)/*(*dε/ε*), where *ε* = *E/k*_*B*_*T* is the reduced energy and *E* is the carrier energy referring to the edge of the corresponding band edge. The scattering can be described by the rate of scattering across an area, measured through the scattered carrier density per unit velocity (*v*) per unit time, i.e., *ξ* = *δN'/δEδVδtδv*. The scattering and transport are reciprocally proportional to each other as *θ ξ* = *D*^2^*v*, where *D* is the density of states at the specific energy. Therefore, the higher the scattering is, the lower the transport. Different transport channels with one or more scattering mechanisms have their own energy sensitivities^43,44^. When multiple channels of transport exist, this energy sensitivity reflects the statistical behavior of the carriers transported within the material system^41^.

Researchers have estimated the average scattering time of carriers by fitting the measured electrical conductivity (*σ*) to a constant relaxation time model^45^ or by observing ultrafast laser-assisted photon-electron interactions^46^. However, there was no well-defined method to extract the value of carrier transport or scattering energy sensitivity from transport measurements until Tang pointed out that the highest achievable Seebeck coefficient could be used to infer this quantity^41^. The Seebeck coefficient characterizes the average entropy carried by each single electron or hole in the semiconducting system microscopically and appears to be the voltage generated per unit temperature gradient in the thermoelectricity production process^47^. Xu et al.^42^ proved that the highest achievable Seebeck coefficient increases linearly with the carrier scattering energy sensitivity in a carbon nanomaterial system once the band structure is fixed. Hence, we can tune the Fermi level through a gate voltage to measure the Seebeck coefficient and then use its highest value to infer the carrier transport energy sensitivity. The strong gate effect of nanoscale materials also ensures the observation of the highest Seebeck values within the typical voltage range of a power supply.

As illustrated in the Supplementary Material, we can plot a map between the highest achievable value of the Seebeck coefficient (*S*_*m*_), the energy sensitivity (*s*), and the band gap (*E*_*g*_) once the asymmetry ratio of transport (γ) between the electrons and holes is determined. Therefore, after we obtain the experimentally measured values *S*_*m*_ for both the *n*- and *p*-type regions of the carbon nanotube network, as well as the corresponding electrical conductivities to calculate γ, we can pinpoint the values of *s* and *E*_*g*_ using this map.

**Figure 1:**
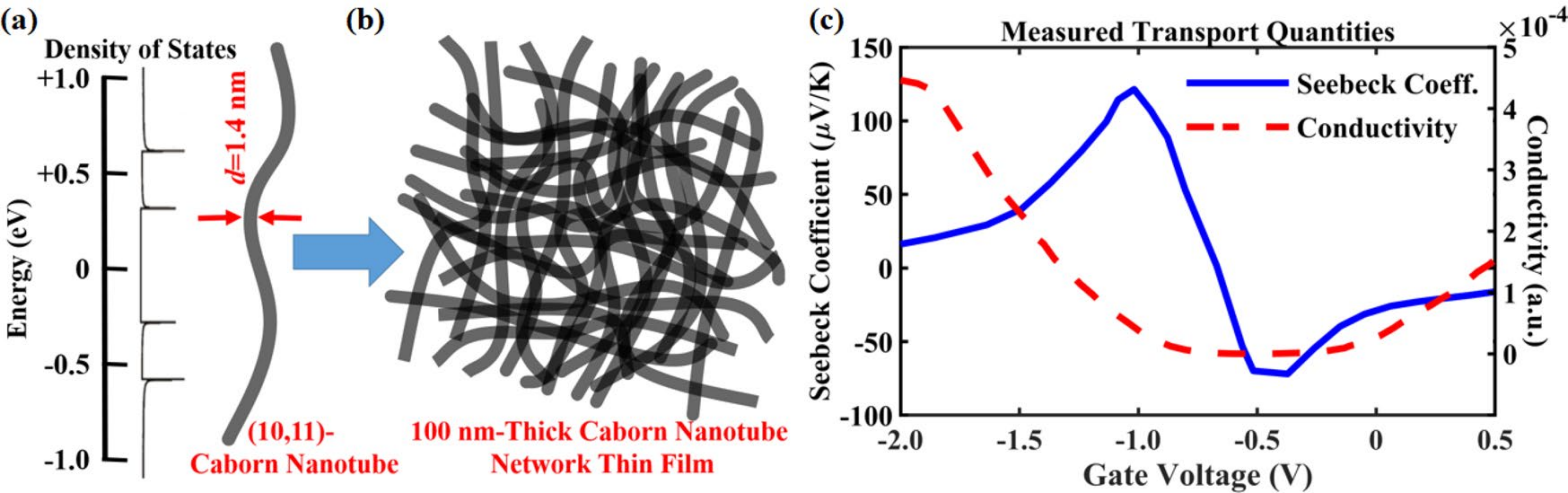
The 100 nm thin film of single-wall carbon nanotube network is synthesized and characterized by Yanagi et al.^48^ The major chirality is of (11,10), corresponding to a diameter of 1.4 nm and a band gap of 0.535 eV. **(a)** The band structure of such a single carbon nanotube is clear. The electronic density of states is showing the peaks caused by van Hove singularities and hence marks the band edges of the semiconducting single-wall carbon nanotube. The first band edges for electrons and holes are marking the band gap, and the second band edges are 0. 260 eV and 0.268 eV apart from the bottom of conduction band and the top of valence band, respectively. **(b)** The band structure or band gap of the carbon nanotube network is not well investigated. The finite distribution of chiralities, imperfections within each nanotube, and the contact points between nanotubes may result in shift and smearing of the effective band edges for transport of this complex materials system. **(c)** The electrical conductivity (dashed red) and the Seebeck coefficient (solid blue) are experimentally measured by Yanagi et al.^48^ at 300 K as a function of Fermi level.

The data of the Seebeck coefficient as a function of gate voltage are measured by Yanagi et al.^48^ for a network of single-wall carbon nanotubes. The majority of the single-wall carbon nanotubes have a chirality of *(11,10)* with a band gap of 0.535 eV, as shown in Fig. 1a. A network with a thickness of 100 nm was fabricated through purification procedures using density gradient centrifugation from single-wall carbon nanotubes produced by the arc-discharge method^49–51^, as shown in Fig. 1b. An electric double layer transistor setup using an ionic liquid as the electrolyte was used to control the Fermi level of the material system by varying the gate voltage.

## Results and discussion

First, it has been difficult to infer the transport band gap from either electrical conductivity or capacitance profiles, since a small density of states near the band edge will not affect these quantities significantly. Researchers have debated whether carbon nanotube network samples should have a band gap similar to that of a single nanotube, e.g., *E*_*g*_ = 0.535 eV for the *(11,10)*-carbon nanotubes in this case. The electrical conductivity and capacitance profiles, as a function of Fermi level measured by Yanagi et al.^48^, do not show an obvious disagreement. However, it is still difficult to explain the small Seebeck coefficient in such a low-dimensional semiconducting material system (not even exceeding ~ 10^2^ μV/K), as shown in Fig. 1c, compared to the fact that pristine graphene with a zero-band gap can achieve a Seebeck coefficient as high as 145 μV/K (*n*-type) and 182 μV/K (*p*-type)^52^, and other two-dimensional materials, e.g., layered silicene and germanene, with a comparable band gap of ~ 0.5 eV can have a Seebeck coefficient on the order of ~ mV/K^53–55^.

**Figure 2:**
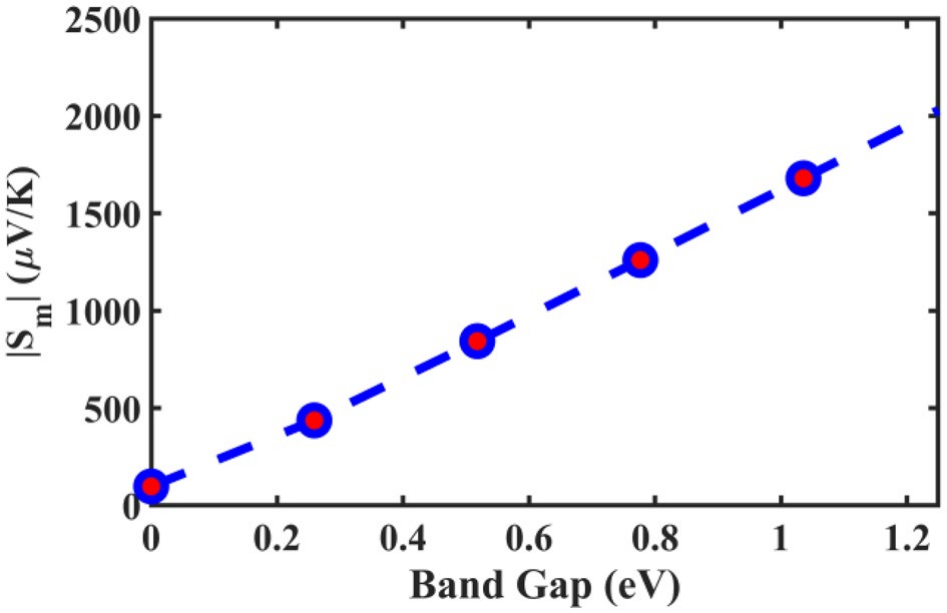
Illustration of how the highest achievable Seebeck coefficient increases with the band gap. Suppose we have a hypothetical carbon material, in which we can fix the mechanism(s) of charge carrier transport and scatterings at *T* = 300 K, and only change the band gap. Specifically, we fix the energy sensitivity to transport at *s* = 1.0 and the asymmetry of transport rates between the holes and the electrons at *γ* = 1.5. This is a demonstration on how the maximum Seebeck coefficient (*p*-type) monotonically increases with the band gap.

The overall Seebeck coefficient of a material system tends to increase with the band gap due to the decreased bipolar effect that causes mutual cancellation between the Seebeck coefficients contributed by electrons and holes. An illustration of how different values of the highest achievable Seebeck coefficient correspond to different band gaps is shown in Fig. 2 for a carbon system with an energy sensitivity to transport *s* = 1.0 and an asymmetry of transport rates between the holes and the electrons of *γ* = 1.5.

**Figure 3:**
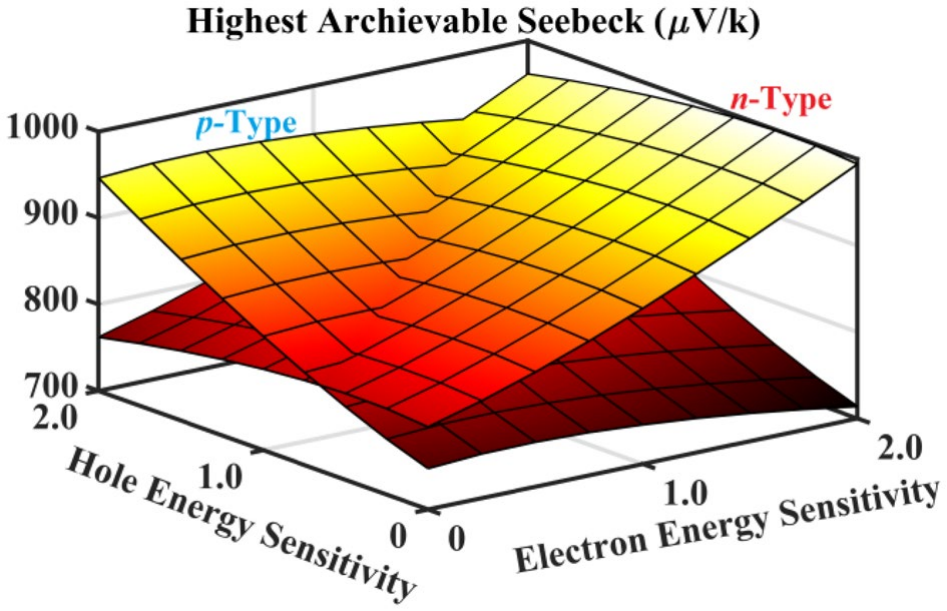
The highest achievable Seebeck coefficient at 300 K is obtained and exhibited for an ideal carbon nanotube network materials system with *E*_*g*_ = 0.535 eV, as a function of energy sensitivities to electron/hole transport. It can be seen that the achievable Seebeck coefficient can be as high as in the order of ~ mV/K, instead of ~ μV/K in the measured data. This means that the band gap of the network for transport could be metallic, instead of semiconducting as expected by some early researchers. It also demonstrates that the highest achievable Seebeck coefficient for the *p*-type and the *n*-type regimes are monotonically increasing and approximately linear with their energy sensitivities to carrier transport. Matlab R2020a (www.mathworks.com) is used to plot this figure, with the license number of 40,836,472.

In Fig. 3, we have calculated the highest achievable Seebeck coefficient in both the *p*-type and the *n*-type regions with different energy sensitivities for electrons and holes, respectively, if the network has the same band gap as a single *(11,10)*-carbon nanotube. It is obvious that the highest Seebeck coefficient would have been much higher than those we obtained from the measured sample. This further implies that the network material cannot have as large an effective band gap for transport as some researchers were expecting.

Now, we can use the highest values of the Seebeck coefficient to infer the band gap, which is much more sensitive to the band edge positions. The positions of the highest achievable values in the curve of the Seebeck coefficient against gate voltage are pinpointed at (−0.02 V, 122.0 μV/K) and (0.63 V, −78.1 μV/K) for this sample. This leads to *E*_g_ = 2.70 meV for the transport band gap, which is much smaller than the thermal energy of *k*_*B*_*T*. Such a band gap value suggests that the network material is effectively metallic at room temperature, which explains the small Seebeck coefficient that is observed. The metallicity of the network material might be due to the wide distribution of diameters and chiralities^32,33^ of the carbon nanotubes within the complex material. A further comparison experiment on the controlled distribution of nanotube diameters is proposed.

**Figure 4:**
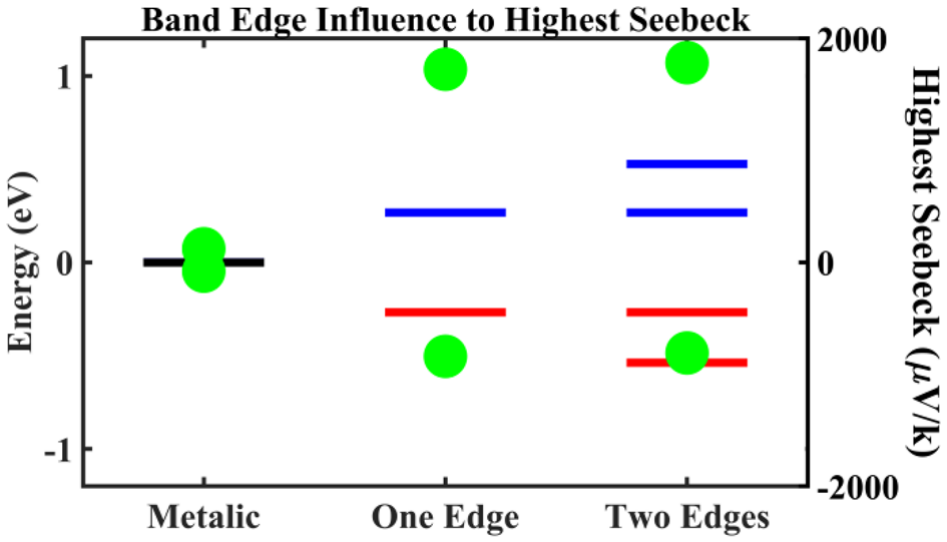
How the position and number of band edges are influencing the highest achievable Seebeck coefficient in a carbon nanotube network system. The first column specifies when the carbon nanotube network is metallic as in the measured sample, the Seebeck coefficient can only be as high as ~ 10^2^ μV/K at 300 K for both p-type (positive value) and n-type (negative). The second column shows that the values of Seebeck can achieve ~ 10^3^ μV/K when the band gap is 0.535 eV and only the first band edge, for either electrons or holes as marked by the van Hove singularity peaks in Fig. 1a, is participating in the transport. The third column shows the highest achievable Seebeck coefficient when the second band edge, for electrons or holes as also marked by the van Hove singularity peaks in Fig. 1a, is participating in the transport. It can be seen that the position has much more significantly influence on the Seebeck coefficient than the number of higher-energy band edges that contribute to the transport.

Before we discuss the energy sensitivity to carrier transport, we first illustrate how sensitive the highest achievable Seebeck coefficient is to the position of band edges of electronic energy. Figure 4 illustrates the highest achievable Seebeck coefficient for three hypothetical carbon nanotube networks. The carrier scattering mechanism(s) are the same for the three networks, and the only differences are the band edges that contribute carriers to the transport. The values of *E*_*g*_ = 0.535 eV and *E*^*’*^_*g*_ = 1.065 eV correspond to the energetic separations between the first and second pairs of van Hove singularities, respectively, within an individual *(11,10)*-carbon nanotube entity, as shown in Fig. 1a. If the band gap of the network is similar to the value of a single *(11,10)*-carbon nanotube, the highest achievable Seebeck coefficient would have been on the order of ~ 10^3^ μV/K for both the *p*-type and the *n*-type regimes. Even if the second pair of van Hove singularities contribute to transport, the highest achievable Seebeck coefficient will still have been on the order of mV/K. This further shows that the present sample of the network is indeed metallic rather than as semiconducting as a single *(11,10)*-carbon nanotube.

The pinpointed maximum points in the Seebeck coefficient curve also infer that the energy sensitivities of carrier transport are *s*_*e*_ = 1.08 for electrons and *s*_*h*_ = 1.10 for holes. At 300 K, electrons in graphene will be scattered strongly by long-range disorders such as thermal ripples^56^, which results in a value of energy sensitivities (*s*_*e*_ and *s*_*h*_) notably greater than 1.00^41,42^. This does not happen here in the carbon nanotube network. The *s*_*e*_ and *s*_*h*_ are still close to 1.00, which might be because the carbon nanotube networks, as well as other complex multiphase nanostructured material systems, are short-range ordered but long-range disordered. Therefore, the phenomenon that long-range thermal ripples interact with block waves is not obviously observed here.

We intuitively assume that the electrons and holes will be strongly resisted during transport through the internanotube contact points, of which the quantum tunneling of electrons is much less efficient than transport within a single carbon nanotube. However, it is interesting to see that the energy sensitivities are also notably greater than 0, which means that the point contact scatterings are not dominant. Alternatively, we can say that the intrananotube resistance is strong enough to make the internanotube resistance less obvious. The close to "1" sensitivities suggest that the major scattering mechanism(s) inside a single carbon nanotube can be either inelastic or ballistic. Researchers have found that polarized optic phonons are not active enough at 300 K to strongly scatter the electrons. Furthermore, the average diameter of the carbon nanotubes in this network sample is believed to be ~ 1.4 nm^48^, which is comparable to or smaller than the mean free path of electrons scattered inelastically. Therefore, we can speculate that ballistic scattering inside the single carbon nanotubes is the dominant mechanism at this temperature inside this sample. The asymmetry of transport between holes and electrons (γ) is inferred to be 1.75, which is similar to the value of 1.50 observed in graphene samples at the same temperature^41,42^.

## Conclusion

Although important for most applications of electronics, energy, and sensing, it has been a long-term challenge to detect the energy sensitivity of carrier transport under external fields and to infer the transport band gap for complex networks consisting of multiple single-phase nanoscale entities.

We have proposed that these quantities can be inferred by measuring the highest achievable Seebeck coefficient by tuning the Fermi levels and have applied this methodology in a network material system assembled with semiconducting carbon nanotubes at 300 K. The following has been found. (1) The network exhibits metallic transport behavior, despite the semiconductivity and finite band gap of a single carbon nanotube. This metallicity is consistent with the small values of the Seebeck coefficient observed in the experiment. (2) Long-range disorder-induced scattering, such as thermal ripples, is suppressed by long-range randomness. (3) Contact point-induced scattering appears to have a minor effect compared to intrananotube ballistic scattering, which is consistent with the small average diameter of 1.4 nm that is observed. (4) The transport asymmetry ratio between the holes and electrons is 1.75, which is consistent with the value of 1.50 observed in pristine graphene samples at the same temperature.

In addition to carbon nanotube networks, this methodology can also be used in other complex material systems, including networks, matrices, and thin films consisting of nanotubes, nanoribbons, nanowires or nanoparticles.

## Supplementary Information

Supplementary Information.
