# Supplementary material for: Inferring the energy sensitivity and band gap of electronic transport in a network of carbon nanotubes
Source: Sci Rep. 2022 Feb 8;12:2060. doi: 10.1038/s41598-022-06078-x (PMC8826413; doi:10.1038/s41598-022-06078-x)
Supplement: Supplementary file 1 — Supplementary Information. [file 41598_2022_6078_MOESM1_ESM.docx]

**Inferring the Energy Sensitivity and Band Gap of Electronic Transport in a Network of Carbon Nanotubes**

Shuang Tang*^a^

^a^ College of Engineering, State University of New York, Polytechnic Institute, Albany/Utica, New York, 12203/13502, USA. E-mail: [tangs1@sunypoly.edu](mailto:tangs1@sunyit.edu)

**SUPPLEMENTARY MATERIALS**

**1. More Details about the Transport Function and the Scattering Rate**

The transport can be described by the rate of transport (*θ*) that measures the density of charge carriers transported within the material per unit time, i.e. $\theta=\frac{\delta N}{\delta E\delta V\delta t}$, where *N*, *E*, *V* and *t* stand for the number of carriers, energy, volume and time, respectively. The scattering can be described by the rate of scattering across an area, measured with the scattered carrier density per unit velocity (*v*) per unit time, i.e. $\xi=\frac{\delta N^{'}}{\delta E\delta V\delta t\delta v}$. The scattering and the transport are reciprocally proportional to each other as $\theta\xi=D^{2}v$, where *D* is the density of states at the specific energy. Therefore, the stronger the scattering is, the smaller transport we have. The transport rate function *θ* can be described by either the relaxation approximation using the Boltzmann equation or the iterative methods to take into consider both the elastic and the inelastic scatterings, e.g. the Rode’s method etc. ^[1-9](#_ENREF_1)^ If the probability distribution of electrons in the momentum space is written as ^[10](#_ENREF_10)^,

$f\left( \boldsymbol{k} \right)=f_{0}\left[ \varepsilon\left( \boldsymbol{k} \right) \right]+xg\left( \boldsymbol{k} \right)$, (Eq. S1)

where *f* is the probability distribution function of electrons in the **k** space, and *f_0_* is *f* function when the system reaches equilibrium, i.e. the Fermi-Dirac distribution, *x* is the angle between the momentum **k** and the externally exerted force, *g*(**k**) is the perturbation to the distribution. The perturbation *g*(**k**) now includes both the inelastic and elastic scatterings, as

$g\left( \boldsymbol{k} \right)=\frac{\chi_{i,in}\left[ g\left( \boldsymbol{k} \right) \right]+\chi_{e,in}\left[ g\left( \boldsymbol{k} \right) \right]+\chi_{b,in}\left[ g\left( \boldsymbol{k} \right) \right]+\chi_{h,in}\left[ g\left( \boldsymbol{k} \right) \right]-v\left( \boldsymbol{k} \right)\left( \frac{\partial f}{\partial z} \right)-\frac{e\boldsymbol{\varepsilon}}{\hbar}\nabla_{\boldsymbol{k}}f}{\chi_{i,out}\left( \boldsymbol{k} \right)+\chi_{e,out}\left( \boldsymbol{k} \right)+\chi_{b,out}\left( \boldsymbol{k} \right)+\chi_{h,out}\left( \boldsymbol{k} \right)}$, (Eq. S2)

where the *χ_in,*_* and *χ_out,*_* terms are counting the probability increment caused by inward and outward transport, respectively. The subscripts of "*i*", "*e*", "*b*", and "*h*" stand for the inelastic, elastic, ballistic and hopping transport, respectively. The term $\left( \frac{\partial f}{\partial z} \right)$ is counting the change of probability density caused by the temperature gradient along the z direction, and the term $\frac{e\boldsymbol{\varepsilon}}{\hbar}\nabla_{\boldsymbol{k}}f$ is counting the effect of external electrical fields. This expression is in a self-involved manner, and can be solved through iterations until convergence.

**2. Band Gap Distribution of Carbon Nanotubes**

The I-V curve can be used as another evidence for the metallic property of the carbon nanotube networks. The I-V curves at different temperatures have been measured by researchers ^[11](#_ENREF_11)^, in which the linearity is confirmed. Unfortunately, it is still challenging to synthesize a carbon nanotube network formed by carbon nanotubes with a single value of diameter/band gap. There usually exist a distribution of nanotube diameter, and thus, a distribution of nanotube band gap, as measured by Zhang et al.^[12](#_ENREF_12)^, who also confirmed the mixture band gaps using Raman spectra shift.

**3. Influence of Higher-Energy Band Edges**

Since the rule of calculating the overall Seebeck coefficient (*S_overall_*) from Band Edges 1 and 2 is ^[13](#_ENREF_13)^:

$S_{overall}=\frac{S_{1}\sigma_{1}+S_{2}\sigma_{2}}{\sigma_{1}+\sigma_{2}}=\frac{\frac{S_{1}}{\sigma_{2}}+\frac{S_{2}}{\sigma_{1}}}{\frac{1}{\sigma_{1}}+\frac{1}{\sigma_{2}}}$, (Eq. S3)

where *S_1_* (*σ_1_*) and *S_2_* (*σ_2_*) stand for the Seebeck coefficient (electrical conductivity) of Band Edges 1 and Band Edges 2, respectively. If we assume that Band Edges 1 is lower in energy and Band Edges 2 is higher in energy, as illustrated in Figure S1


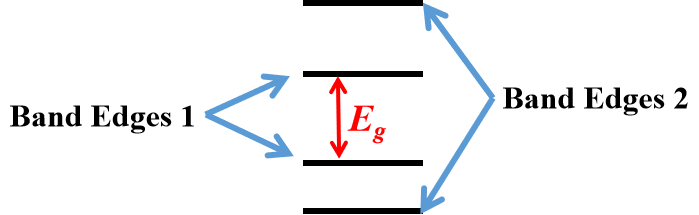


**Figure S1:** Scheme of Band Edges 1 and Band Edges 2. The separation in energy between the Band Edges 1 is the band gap.

Because higher bands will contribute less in electrical conductivity, it is obvious that *σ_1_* > *σ_2_,* so the dominating Seebeck coefficient comes from Band Edges 1. If *E_g_* is large, though *σ_1_* > *σ_2_*, yet *σ_2_* may still be comparable with *σ_1_*, so counting the contribution from Band Edges 2 will noticeably affect the overall Seebeck; if *E_g_* tends to be 0, *σ_1_* >> *σ_2_*, whether or not including *S_2_* in calculating *S_overall_* will not have noticeable differences. In our case, only when the effective band gap of the carbon nanotube network tends to be zero, the higher-energy band edges will not have noticeable influence on the calculation of the overall Seebeck coefficient.

**4. How the highest achievable Seebeck coefficient is determined by the energy sensitivity and band gap at a specific temperature.**

Once the asymmetry ratio (*γ*) between the *n-* and *p-*type electrical conductivities corresponding to the maximum Seebeck coefficients (*S_m_*) are obtained, we can determine the energy sensitivity and the band gap from the measured values of *S_m_*. For example, for an asymmetry ratio of 1.5, the mapping between the *S_m_*, the energy sensitivity and the band gap for a carbon nanotube network system is shown in Figure S2. If we know the values of *S_m_*, the energy sensitivity and the band gap can be inferred from this map.


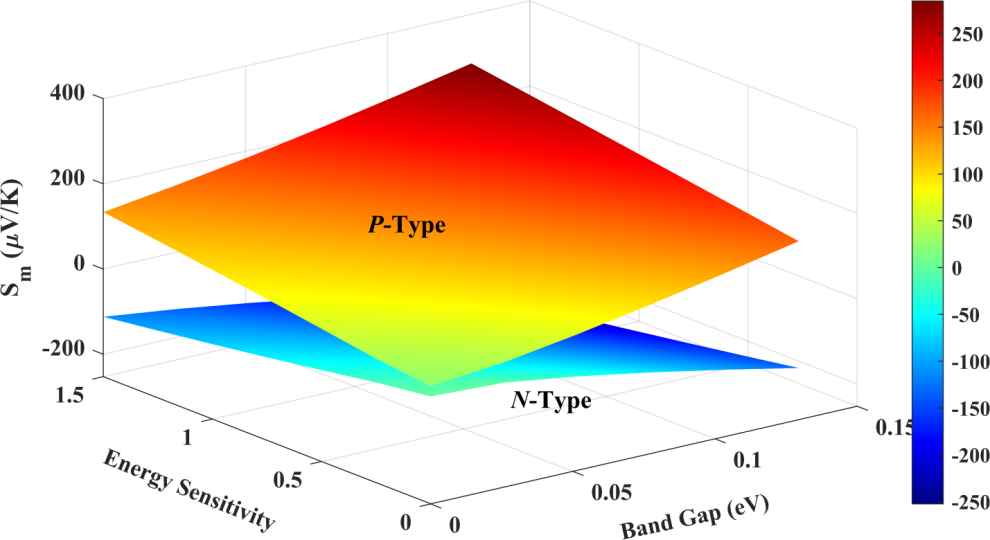


**Figure S2:** The mapping between the highest achievable Seebeck coefficient for the *p-*and *n-*type regions, the energy sensitivity of carrier transport, and the band gap for a carbon nanotube network system. Matlab R2020a (www.mathworks.com) is used to plot this figure, with the license number of 40836472.

**REFERENCES:**

1 Rode, D. in *Semiconductors and semimetals* Vol. 10 1-89 (Elsevier, 1975).

2 Miller, N. *et al.* Effect of charged dislocation scattering on electrical and electrothermal transport in n-type InN. *Physical Review B* **84**, 075315 (2011).

3 Rode, D. Electron mobility in direct-gap polar semiconductors. *Physical Review B* **2**, 1012 (1970).

4 Ng, G., Vasileska, D. & Schroder, D. Calculation of the electron Hall mobility and Hall scattering factor in 6 H-SiC. *Journal of Applied Physics* **106**, 053719 (2009).

5 Lundstrom, M. *Fundamentals of carrier transport*. (Cambridge university press, 2009).

6 Ferry, D. *Semiconductor transport*. (CRC Press, 2000).

7 Ramu, A. T. *et al.* Rigorous calculation of the Seebeck coefficient and mobility of thermoelectric materials. *Journal of Applied Physics* **107**, 083707 (2010).

8 Ramu, A. T. *et al.* Thermoelectric transport in the coupled valence-band model. *Journal of Applied Physics* **109**, 033704 (2011).

9 Ramu, A. T. & Bowers, J. E. The impact of commonly used approximations on the computation of the Seebeck coefficient and mobility of polar semiconductors. *Applied Physics Letters* **101**, 173905 (2012).

10 Faghaninia, A., Ager III, J. W. & Lo, C. S. Ab initio electronic transport model with explicit solution to the linearized Boltzmann transport equation. *Physical Review B* **91**, 235123 (2015).

11 Pöhls, J.-H. *et al.* Physical properties of carbon nanotube sheets drawn from nanotube arrays. *Carbon* **50**, 4175-4183 (2012).

12 Zhang, F. *et al.* Growth of semiconducting single-wall carbon nanotubes with a narrow band-gap distribution. *Nature communications* **7**, 1-9 (2016).

13 Goldsmid, J. *The physics of thermoelectric energy conversion*. (Morgan & Claypool Publishers, 2017).
